# Glycolysis and Automated Plaque Regrowth Method for Evaluation of Antimicrobial Performance

**DOI:** 10.3390/dj12050146

**Published:** 2024-05-17

**Authors:** Robert L. Karlinsey, Tamara R. Karlinsey

**Affiliations:** Custom Dental Formulations, LLC, 1291 Airport Parkway, Suite 400, Greenwood, IN 46143, USA; trk@customdentalformulations.com

**Keywords:** antiplaque, antigingivitis, antimicrobial, chemical plaque control, preventive dentistry, essential oil, zinc, stannous, fluoride, CPC

## Abstract

Purpose: This study explored the potential of a new in vitro method in evaluating antiplaque benefits from five sets of antimicrobial systems including cetylpyridinium chloride (CPC), stannous fluoride (SnF_2_), Listerine essential oil mouthwashes (+/− alcohol), zinc chloride (ZnCl_2_), and sodium fluoride. (NaF). Methods: Gingival dental plaque was collected and propagated using sterilized tryptic soy broth and sucrose, and then allocated into separate glycolysis and regrowth recipes for antiplaque evaluations. Glycolysis measurements (in duplicate) were recorded via pH microelectrode on plaque-treatment samples thermomixed (1200 rpm, 37 °C) for 4 h. For plaque regrowth, optical densities (in duplicate) were automatically collected on plaque-treatment samples using a microplate reader (linear shaking, 37 °C) from baseline to 4 h. Results: Calculations of percent change in pH and optical density were performed and analyzed for each set of antimicrobial treatment groups. Statistical analysis (one-way ANOVA, Student–Newman–Keuls stepwise comparison tests) revealed dose responses and significant differences (*p* < 0.05) among treatment groups, including between negative and clinically relevant positive controls. Conclusions: This lab method produces results consistent with published clinical observations. This glycolysis and plaque growth method is sensitive to antimicrobial mechanisms of action, and may offer a convenient and clinically relevant screening tool in the evaluation of putative antimicrobial agents and formulations.

## 1. Introduction

The global prevalence of dental caries and periodontitis continues to increase [1]. With respect to the latter, as reported by the European Federation of Periodontology and the World Heart Foundation, positive association exists between periodontitis and coronary heart disease [2]. While a case–control study found increased risks of periodontitis in obese adolescents [3], the natural aging process introduces greater periodontitis risks—risks that are magnified in populations with caries experience [4]. Thus, if managing periodontal disease is one way to improve dental, and possibly overall health, there remains strong interest in identifying, formulating, and evaluating potential antimicrobial systems in economical, efficient, and meaningful ways.

Randomized clinical trials (RCT) remain the gold standard for confirming (or negating) an ingredient or formulation. Due to economic or efficiency reasons, screening or quality evaluations can be performed using relevant laboratory models sensitive to negative and positive controls. With respect to evaluations of potential antimicrobial agents or formulations, typical minimum inhibition concentration (MIC) and kill-time laboratory assays provide initial insight into potential antimicrobial activity [5,6]; however, these do not provide bioavailability or retention information, both of which can strongly influence the likelihood of clinical success [7,8]. Notably, these lab-based studies can be subject to protocol variations which can infuse additional uncertainty: for example, there exist conflicting reports on the potential antimicrobial activity of theobromine [9,10,11,12,13,14].

The introduction of the plaque glycolysis and regrowth method (PGRM) in the mid-1990s by Procter & Gamble was part of the evaluation of a new stannous fluoride dentifrice and provided an approach that might be used as a surrogate to clinical outcomes [7]. The ex vivo PGRM model probes antiglycolytic and microbial regrowth activity of a volunteer’s plaque obtained by freshly swabbing the gingival margin pre- and post-treatment (i.e., human or animal intervention). In contrast, in vitro PGRM models utilize banked and propagated plaque instead of immediate-use harvested plaque (i.e., no direct human or animal intervention). Because PGRM observations yielded reasonable agreement with clinical outcomes for some well-known antimicrobial agents (e.g., stannous fluoride, cetylpyridinium chloride, and chlorhexidine gluconate), it now serves as a clinically relevant screen for new antiplaque/antigingivitis formulations. For example, the United States’ Food and Drug Administration issued testing guidance for over-the-counter antigingivitis/antiplaque formulations and included the use of PGRM methods for evaluating the biological activity of formulations containing stannous fluoride (SnF_2_) or cetylpyridinium chloride (CPC) [15]. Similarly, the American Dental Association recognizes the utility of PGRM models in their evaluation of dental products for the antigingivitis seal of acceptance [16].

Notably, there does not exist a sole PGRM model that is used to uniformly assess potential antimicrobial activity. As such, variations and improvements in these models can be expected. While human plaque and the use of thermomixing was used in the original publication [7], recently, Procter & Gamble published in vitro PGRM data that utilized glass rods and human saliva-derived oral microbes to show promising antiglycolytic benefits of hops beta acids [17]. In this paper, we describe a modified PGRM model that we refer to as glycolytic and automated plaque regrowth (GAPR). Our GAPR model maintains the use of thermomixers in obtaining antiglycolytic data but also utilizes an automated microplate reader to record optical density.

The original PGRM method utilized a thermomixer but required aliquots to be removed from the parent vial and dispensed into an analog microplate reader for optical density measurements over the course of, for example, two or four hours (with measurements taken intermittently) [7]. Such removal introduces potential errors including human, thermal, and volume-based errors, especially if the treatment-plaque mixture is inhomogeneous (e.g., essential oils naturally mix poorly with water-based plaque and saliva samples).

In using an automated microplate reader to measure optical density in a closed system at constant temperature and mixture volume, we greatly reduce, if not eliminate, these experimental errors. Furthermore, we have identified treatment–plaque media ratios that allow for the evaluation of other known clinically proven antimicrobial active ingredients beyond stannous fluoride and cetylpyridinium chloride, including zinc chloride (ZnCl_2_), essential oil combinations (e.g., Listerine^®^), and other systems. We believe this GAPR method significantly contributes to the type of PGRM models available for screening of potential antimicrobial agents and formulations.

In this paper, we describe the evaluation of several familiar antimicrobial systems using the GAPR method and propagated human plaque harvested from the gingival margins. Glycolytic and/or optical density data are presented for systems including CPC solutions, buffered SnF_2_ systems, aqueous sodium fluoride (NaF) systems, ZnCl_2_ solutions, and some commercially available Listerine^®^ essential oil systems. The purpose of using these different antimicrobial systems is to highlight the sensitivity of this GAPR method to different antimicrobial mechanisms of action. This is especially important since PGRM models are not generally used to evaluate antimicrobials beyond CPC and stannous fluoride systems [7,15].

## 2. Materials and Methods

All chemicals and labware detailed below were sourced from Fisher Scientific or other comparable supply houses. Solutions were made using calibrated pipettes and sterilized labware, and reagents and powders were measured using an Ohaus Adventurer Pro AV213 Balance. Sterilization (Moosoo Model No. GT606-M10) was performed on all relevant labware, solutions, and media to ensure plaque-only microbial responses. A Fisherbrand Accumet AB315 pH/mV meter fitted with a Mettler Toledo LE422 pH microelectrode were used to measure pH of all solutions and media. A Barnstead/Thermolyne Cimarec^®^ heat/stir plate and BT Lab Systems multi-position stir plate were used to magnetically agitate solutions and media using Teflon-coated stir bars.

### 2.1. Plaque Harvesting and Propagation

The experimental procedure for this in vitro study utilized pooled human plaque donated from two consenting volunteers (RLK and TRK) with caries experience but having good oral and systemic health. Each volunteer refrained from food, drink (except for water), and oral hygiene for at least 10 h overnight prior to plaque collection. On the morning of collection, sterile cotton swabs were used to harvest the plaque at the buccal and lingual gingival margins (collecting both supra- and subgingival plaque) in each quadrant of the mouth. This was done by wiping the swab from molar to incisor (or incisor to molar). After separating the swab tip from the shank, the swab was then placed into a 7 mL tube containing 1.5 mL of 6% tryptic soy broth (TSB). A cap was placed on the vial to seal it and it was vortexed for 10 s. After vortexing, the contents were dispensed into a 20 mL sterile vessel and the swab was extracted with forceps and disposed of in a sealed biohazard bag. For each volunteer, there were then four vials, totaling 6 mL of plaque–TSB mixture. These were subsequently pooled, resulting in 12 mL of plaque–TSB fluid.

Techniques used for plaque growth are based on published reports [7,8]. Plaque was initially grown by combining the 12 mL of plaque–TSB mixture with 28 mL of additional 6% TSB along with 8 mL of 40% sucrose solution into sterile glass container. The container was then placed into a Fisher Scientific Isotemp Oven at 37 °C. To generate anaerobic conditions, CO_2_ gas was evolved by combining sodium bicarbonate with citric acid and water in a separate vessel and placed in the same oven (0.65 ft^3^). The propagation went overnight (at least 12 h) and produced high-density, acidic plaque media with pH near 4.4 and optical densities of at least 0.8.

Plaque propagation is continued over the course of several days without sacrificing plaque quality and response. This is accomplished by taking 1 mL of the prior day’s plaque as the inoculant and dispensed into a sterilized vessel with 6% TSB (40 mL) and 40% sucrose (8 mL).

### 2.2. Glycolysis and Automated Plaque Regrowth (GAPR) Procedures

Recipes used for glycolysis and plaque regrowth are as follows. From the propagated plaque mixture described above, aliquots were extracted and diluted using 6% TSB for kinetic regrowth or 0.03% TSB for glycolysis experiments. To probe plaque acid production, sucrose solution (40%) was added to glycolysis experiments.

On the morning of evaluation, aliquots of the propagated plaque were extracted and mixed with either 0.03% or 6% TSB according to the desired experiment. In this investigation, we used two-fold dilution for glycolysis experiments using 0.03% TSB, and 8- or 10-fold dilutions for kinetic growth experiments using 6% TSB. For the glycolysis experiments, it was necessary to standardize the diluted plaque to neutral pH, and this was performed using 1M NaOH. No standardization was required for the kinetic growth experiments.

For the evaluation of glycolysis (i.e., fermentation of sucrose) by a given treatment, for every 1 mL of standardized plaque (as described above), 50 µL of 40% sucrose and either 17.5, 50, or 200 µL of treatment (depending on the experiment) were added to an Eppendorf 1.5 mL vial. Each vial was vortexed at high speed for 10 s and then follow-on baseline pH measurements were made. Afterwards, samples were loaded into an Eppendorf Thermomixer set at 37 °C with 1200 rpm agitation and processed for up to 4 h, with periodic interruptions made to measure pH (e.g., at the 2 h mark).

For plaque regrowth experiments, the plaque sample was diluted 8-fold or 10-fold using 6% TSB. Then, either 0.5 mL or 1 mL amounts were used to create the plaque sample. To the 0.5 mL plaque sample, 0.5 mL of treatment was added (1 mL total volume); or, to the 1 mL plaque sample, 5 µL or 50 µL of treatment was added. These treatment–plaque samples were combined in Eppendorf 1.5 mL vials and vortexed at high-speed for 10 s. Afterwards, single aliquots of 200 µL were dispensed into designated wells within a 96-microwell plate, covered, and inserted into an Agilent Epoch 2 microplate reader operating at 37 °C under continuous linear shaking to encourage bacterial growth. Automated kinetic growth experiments were subsequently performed in real time at 600 nm for up to 4 h in 15 min increments. We note this is a significant improvement over prior methods due to increased speed, efficiency, and reduction in possible error (both experimental and volume-induced) [7]. As optical density is related to the concentration and turbidity of microbial species, this is an estimate of both live and dead species, as well as any contribution that may arise from moiety absorption present in the treatment or media: for instance, at 600 nm, which is the chosen wavelength for optical density measurements, CPC contributes modest absorption, while blue dyes (e.g., blue 1) contribute more significantly. This assumption holds true in our method and evaluations as well.

### 2.3. Glycolysis and Regrowth Calculations

**Figure 1:**
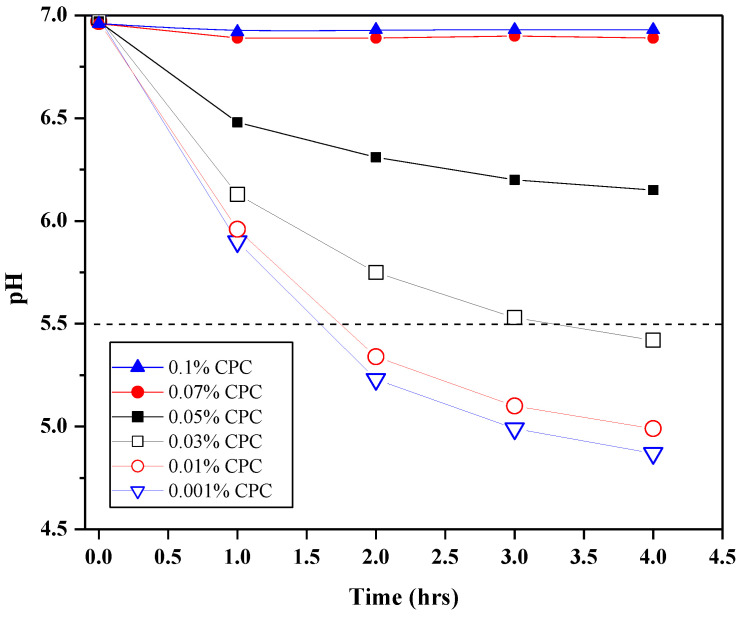
Glycolysis (via pH measurements) produced from human plaque treated with 0.001% (blue open triangles, line), 0.1% (red open circles, line), 0.03% (open black squares, line), 0.05% (closed black squares, line), 0.07% (closed red circles, line), or 0.1% (closed blue triangles, line) CPC, 50 μL sucrose, and thermomixed at 37 °C for up to four hours. The dashed line at pH 5.5 marks the dissolution of enamel.

**Figure 2:**
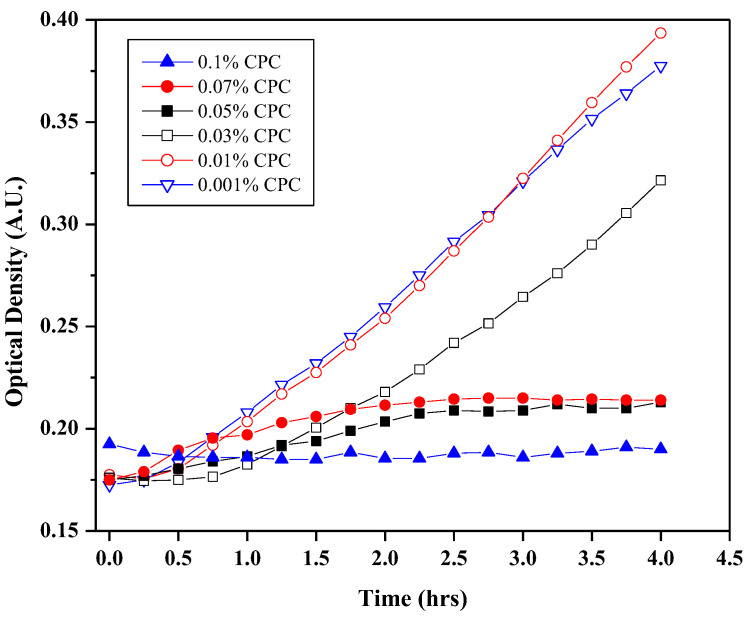
Plaque regrowth (via optical density measurements) produced from human plaque treated with 0.001% (blue open triangles, line), 0.1% (red open circles, line), 0.03% (open black squares, line), 0.05% (closed black squares, line), 0.07% (closed red circles, line), or 0.1% (closed blue triangles, line) CPC at 37 °C for up to four hours.

In general, there are various ways to present glycolysis and regrowth data generated from PGRM evaluations, such as those shown in Figure 1 and Figure 2, including simple differences, area-under-curves, lineshape fittings, percentages, etc. We chose to calculate percent changes, and we processed all data according to the following equations. For glycolysis, pH measurements were made at baseline (*pH_Base_*) and after up to four hours (*pH*_4*hrs*_) of thermomixing, and the mean change in pH at these two timepoints were determined according to Equation (1):

(1) $$\%\;pH=\left(\frac{{pH}_{4hrs}-{pH}_{Base}}{{pH}_{Base}}\right)\times 100.$$

The ability to quickly assess the potential antiglycolysis benefit of a treatment can be accomplished by creating an efficiency parameter, such as percent acid inhibition (Acid Inhibition, %) [17]. In doing so, we calculated the percent acid inhibition for each of the treatments using the average % pH calculated according to Equation (1) for each treatment system. To make this relative comparison, a negative control is used as the reference and is therefore set to 0. Because this acid inhibition parameter is for estimate purposes only, no statistical evaluations were made; however, statistics were performed on the values determined from Equation (1) for a given treatment. Thus, the percent acid inhibition is obtained according to Equation (2):

(2) $$Acid\;Inhibition\;(\%)=100-\left(\frac{{\%\;pH}_{Tx}}{{\%\;pH}_{N.C.}}\right)\times 100,$$

where (*% pH_Tx_*) corresponds to the mean percent change in pH observed for any other treatment besides the selected negative control (e.g., 0.001% CPC). Similarly, (*% pH_N.C._*) corresponds to the mean percent change in pH observed for the negative control. Both mean percent changes are separately calculated using Equation (1). In the event the pH increases above baseline values, which can happen with highly effective antiglycolysis treatments such as 0.1% CPC, then the value is capped at 100.

For plaque regrowth, optical density data were automatically collected at baseline (*OD_Base_*) and then after four hours (*OD*_4*hrs*_) of rapid linear shaking in the microplate reader. After the experiment, these data points were then used to calculate the mean percent change in OD at these two timepoints for each replicate according to Equation (3):

(3) $$\%\;OD=\left(\frac{{OD}_{4hrs}-{OD}_{Base}}{{OD}_{Base}}\right)\times 100.$$

### 2.4. Data Sets and Statistical Analyses

Each pH and optical density measurement was performed in duplicate (an original and a replicate). This means, for a given treatment, there will be six pH measurements (baseline, 2, and 4 h) and at least 16 optical density measurements. These measurements were assessed for descriptive statistics, including means (*n* = 2), standard deviations, and standard error of the mean (SEM). Comparisons among treatments were then performed using a one-way analysis of variance (ANOVA) model using Sigma Plot version 14.5 (Systat Software). If significant differences were detected (*p* < 0.05), then the means were evaluated pairwise using the Student–Newman–Keuls (SNK) test to identify where differences existed.

## 3. Results

### 3.1. Examples of Glycolysis and Regrowth Curves for CPC Solutions

The glycolytic response of plaque when exposed to 17.5 μL of aqueous CPC solutions at 0.001%, 0.01%, 0.03%, 0.05%, 0.07%, and 0.1% is shown in Figure 1. This is an example plot of characteristic responses to inferior (i.e., 0.001% or 0.01% CPC), where the pH drops significantly, or superior (e.g., 0.07% or 0.1% CPC), where the pH maintains minimum drops, performance. The pH of the dissolution of enamel is also included for reference (pH 5.5). This figure clearly reveals the differences among the treatments, and further analysis and details will be given below in Table 1.

An example of the plaque regrowth response when exposed to 5 μL of the same aqueous CPC solutions is shown in Figure 2. In this assessment, plaque regrowth is strong in the absence of a strong antimicrobial agent, as reflected in the inclining slope for 0.001% and 0.01% CPC. In contrast, little regrowth occurs for strong antimicrobial agents, and the growth curve offers minimal increases (e.g., 0.05% or 0.07% CPC), or even decreases (e.g., 0.1% CPC). Like Figure 1, this figure also reveals marked differences among the treatments, and further analysis and details are presented in Table 1.

### 3.2. Glycolysis and Regrowth Response from CPC Solutions

Using three equations in Section 2.3, the performance of 0.001%, 0.01%, 0.03%, 0.05%, 0.07%, and 0.1% CPC is summarized in Table 1. For the glycolysis experiments, the treatments were all the same at 17.5 µL; for regrowth, 5 µL treatments were used. Statistical analyses revealed that significant differences (*p* < 0.05) existed within each respective glycolysis and regrowth column.

For glycolysis, each of the six CPC groups were found to be statistically independent from one another (indicated by the lowercase letters), with 0.001% and 0.1% CPC solutions performing the worst and best at inhibiting fermentation of sugar, respectively. As described above, the acid inhibition metric serves as an efficiently parameter that allows one to quickly ascertain the efficacy of a given treatment. In this evaluation, the best acid inhibition is achieved at the 0.07% and 0.1% CPC levels.

**Figure 3:**
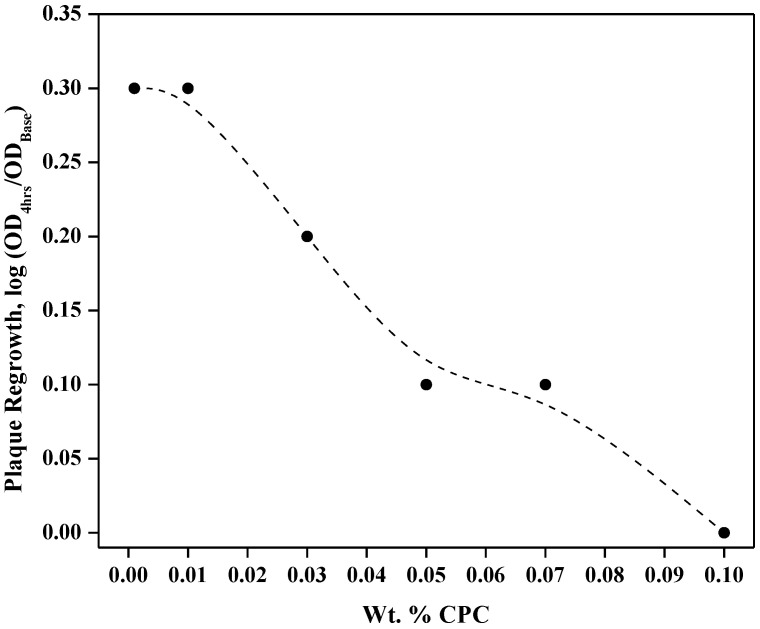
Plaque regrowth response as a function of CPC weight percent, including those recommended for antiplaque/antigingivits benefits.

With respect to plaque regrowth, there were statistical differences; however, 0.001% and 0.01% CPC were found to be statistically similar (*p* > 0.05) but inferior to all other higher-content CPC groups. Likewise, the 0.05% and 0.07% CPC solutions were also similar (*p* > 0.01) but were superior to the lower-content CPC groups and inferior to the 0.1% CPC group. Figure 3 visualizes the plaque regrowth–CPC relationship from the perspective an antimicrobial kill-time curve.

### 3.3. Glycolysis and Regrowth Response from NaF Solutions

Calculations according to the three equations in Section 2.3 were performed in the assessment of antiplaque performance of 0.1 ppm, 1 ppm, 100 ppm, 1000 ppm, 5000 ppm, and 22,500 ppm fluoride (NaF) solutions. Because NaF does not typically deliver antiplaque/antigingivitis benefits, a 0.1% CPC positive control group was also included, along with the 0.001% CPC negative control. The performance is summarized in Table 2. For the glycolysis experiments, the treatments were all the same at 50 µL; for regrowth, 50 µL treatments were also used (including for 0.001% and 0.1% CPC). Statistical analyses revealed significant differences (*p* < 0.05) existed within each respective glycolysis and regrowth column.

For glycolysis, only the 22,500 ppm F group exhibited the largest inhibition of acid production among NaF groups, with all other groups exhibiting poor antiglycolytic potential. Among these groups, significant differences were detected for all groups except 0.1 ppm, 1 ppm, and 100 ppm F, which were found to be statistically similar. The least effective antiglycolysis group was the 0.001% CPC negative control. The positive control, 0.1% CPC, produced the greatest antiglycolytic activity and, in turn, produced the highest acid inhibition.

**Figure 4:**
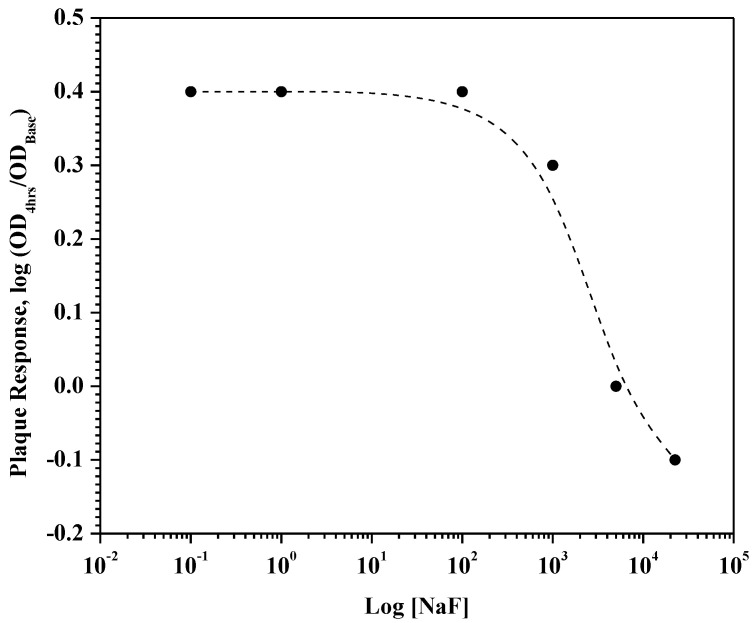
Plaque regrowth response as a function of NaF content. The range of NaF shown covers those levels in community water supplies (e.g., 0.1 ppm F) up to varnishes with 5% NaF.

With respect to plaque regrowth, statistical analysis revealed 22,500 ppm F inhibited growth better than the 0.01% CPC control, which was statistically similar to 5000 ppm F. Similar to glycolysis, the 0.1 ppm, 1 ppm, and 100 ppm F groups were statistically similar and produced the largest plaque regrowth, even greater than 0.001% CPC. The 1000 ppm F group delivered statistically better regrowth control relative to 0.001% CPC, and was significantly different relative to the lower fluoride levels, as well as the higher fluoride levels. To help illustrate the difference between the antimicrobial sensitivity of CPC compared to fluoride, a plaque regrowth–NaF relationship from the perspective an antimicrobial kill-time curve was created (Figure 4).

### 3.4. Glycolysis and Regrowth Response from SnF_2_ Citrate Buffered Solutions

Separate GAPR experiments were also conducted to evaluate the effect of citrate buffers with and without SnF_2_ and pH 3.8 and 6. Using the same equations in Section 2.3, antiplaque performance of the four citrate buffered solutions was assessed and summarized in Table 3. Due to the expected, and then observed, poor performance of the pH 6 citrate buffer, this was selected as the negative control. For the glycolysis experiments, the treatments were all the same at 50 µL; for regrowth, 50 µL treatments were also used. Statistical analyses revealed significant differences (*p* < 0.05) existed within each respective glycolysis and regrowth column.

All groups were statistically different from one another with respect to glycolysis and plaque regrowth. The citrate buffer at pH 3.8 with 0.4% SnF_2_ produced the largest inhibition of acid production and smallest plaque regrowth among the four groups. These results were significantly different from all other groups, including the citrate buffer at pH 6 with 0.4% SnF_2_. For each buffered system, the statistical differences show the presence of stannous fluoride significantly boosted the antiplaque potential.

### 3.5. Glycolysis and Regrowth Response from Listerine^®^ Mouthwashes

Antiplaque performance of Listerine^®^ essential oil mouthwashes (Johnson & Johnson Consumer Healthcare, Skillman, NJ, USA) are summarized in Table 4. Using the same equations in Section 2.3, the results show distinct differences between the two mouthwashes, with the Listerine^®^ Original^®^ with alcohol (A) exhibiting statistically different glycolysis and regrowth performance relative to Listerine^®^ without alcohol (B). For the glycolysis experiments, we purposefully increased the treatments to 200 µL (instead of 50 µL) for all groups to ensure we did not ‘miss’ a potential benefit; in fact, treatments at lower levels (e.g., 100, 50, or 25 µL) failed to show antiglycolysis potential. For regrowth experiments, the ratio of plaque sample to treatment was fixed at 1:1 (e.g., 0.5 mL plaque sample: 0.5 mL treatment).

In this evaluation, we used mineral oil, which is not known to exhibit antiplaque benefits, as the negative control for regrowth experiments; however, we maintained 0.001% CPC as the negative control for the glycolysis evaluations. We did not use 0.001% CPC as the negative control for regrowth because at 0.5 mL treatment at 1:1 ratio, the CPC content would not be sufficiently dilute to consider it a negative control. We further note that the regrowth performance of the untreated plaque produced 71.1 (6.5) % OD regrowth and was comparable to mineral oil.

The Ultraclean^®^ version (B) contains eucalyptol, zinc chloride, benzoic acid, methyl salicylate, thymol, and menthol. The Listerine^®^ Original^®^ Cool Mint has 21.6% alcohol plus the four essential oils: 0.092% eucalyptol, 0.042% menthol, 0.064% thymol, and 0.06% methyl salicylate. Between these two mouthwashes, the Ultraclean^®^ appears to convey better antiplaque performance, and this could be due to the presence of additional ingredients (e.g., benzoic acid and zinc chloride), changes in content of the four essential oils, or a combination of these.

Statistical analyses revealed that significant differences (*p* < 0.05) existed within each of the groups within each respective glycolysis and regrowth column. While prior evaluations used lower treatment levels for both glycolysis and regrowth evaluations, the poor antiglycolytic results in Table 4 demonstrate that increasing the treatment volume does not necessarily lead to favorable antiplaque performance.

### 3.6. Glycolysis and Regrowth Response from ZnCl_2_ Solutions

Antiplaque performance of ZnCl_2_ solutions, relative to 0.1% and 0.001% CPC, are summarized in Table 5. Again, using the same equations as in Section 2.3, the results show distinct differences among the four groups, with the 0.1% and 0.001% CPC exhibiting statistically superior and inferior performance, respectively, relative to all groups. For the glycolysis experiments, the treatments were all the same at 50 µL. For regrowth experiments, the ratio of plaque sample to treatment was fixed at 1:1 (e.g., 0.5 mL plaque sample: 0.5 mL treatment), the same as the ratio used for the Listerine^®^ mouthwash evaluations.

Statistical analyses revealed significant differences (*p* < 0.05) existed within each of the groups within each respective glycolysis and regrowth column. Here, we used 0.001% CPC as the negative control for both glycolysis and regrowth evaluations. This group performed the poorest and was selected as the negative control baseline for the acid inhibition calculations. Among the four groups, 0.1% CPC provided the greatest acid inhibition. The plaque regrowth data show a statistically significant dose response between 0.08% and 0.2% ZnCl_2_. Both of these zinc chloride solutions were statistically greater than 0.001%, while 0.2% was statistically similar to 0.1% CPC. Between these two groups, 0.1% CPC delivered a dual-action benefit (consistent with the other CPC evaluations described in this paper), while 0.2% zinc chloride only provided meaningful antigrowth benefits.

## 4. Discussion

The determination of how much treatment to administer strongly depends on the putative mechanism of action of the antiplaque/antigingivitis agent. We have found that if an agent (e.g., distilled water, mineral oil, very dilute concentrations of known actives, etc.) lacks the ability to inhibit glycolysis or growth, the volume of treatment matters less. However, if an agent exerts antimicrobial responses, to minimize off-target effects or side-effects, it is reasonable to use the minimum amount needed to induce the antimicrobial response (e.g., antibiotic or vaccine therapy). Through numerous evaluations, we identified a sensitivity of the treatment dose based on the putative mechanism of action of the antimicrobial agent. In other words, actives like CPC, which readily attach to microbial membranes through a combination of ionic and lipophilic interactions [18], fundamentally differ in how they deliver antiplaque benefits relative to say, essential oils (via lipid diffusion and disruption [19]) or stannous fluoride (via the coordination of Sn^2+^ [20]).

The glycolysis and regrowth results reported here on the antimicrobial effects of CPC on plaque are consistent with what has been observed clinically [18,21,22,23,24], and is in line with the FDA Plaque Subcommittee’s recommendation for antigingivitis formulations when formulated with CPC between 0.045% to 0.1% [15]. As shown in Figure 3, the plaque regrowth–CPC content response resembles typical antimicrobial activity observed in kill-time curves [25]. Our estimate that 0.03% CPC inhibits only 26% of acid production, while 0.05% CPC inhibits more than twice this at 61%, appears consistent with guidance put forth by the FDA. Not only are these results important from a validation perspective, but this is also an important formulation aspect, as the retention and bioavailability of CPC remain paramount to its efficacy [8,18,23].

Fluoride remains the gold standard for anticaries but generally does not provide sufficient antiplaque/antigingivitis benefits at low enough levels to support ad libitum over-the-counter use [26,27]. While inhibition of sugar uptake and/or acid production has been shown in lab studies on individual organisms (as low as 1 ppm F) [27,28,29,30,31,32], fluoride’s action on dental plaque remains modest at best and if fluoride is recommended then typically stannous fluoride is used due to the antibacterial capability of the stannous ion [7,20,26,33,34,35]. However, clinical reductions in plaque and/or gingivitis have been observed from formulations comprising much higher concentrations of sodium fluoride (e.g., 2%, 2.26%, or 5% NaF) [36,37] and these plaque glycolysis and regrowth results appear consistent with published observations.

Glycolysis and regrowth experiments were also conducted on two citrate buffer solutions at pH 3.8 or 6, with and without SnF_2_. These citrate solutions are test formulations, and the purpose of the different pH levels was to underline the sensitivity of stannous fluoride to pH, which directly affects Sn^2+^ availability. We note that these stannous fluoride systems are not optimized for performance but were prepared to show how these systems could perform in this glycolysis-regrowth model; in fact, formulation instability might explain the lackluster SnF_2_ gel clinical performance in an evaluation of biofilms on orthodontia [38]. For systems with a pH less than 4, the stannous ion retains its 2+ oxidation state; in contrast, at higher pH (e.g., pH 6), the oxidation state changes to 4+, and this is a less effective antimicrobial form of stannous [20,39]. Separately, sodium citrate and citric acid are chelating systems known to sequester metal ions (e.g., stannous ions [40]), as well as provide buffering benefits for the promotion—or inhibition—of (bio)chemical reactions [41]. Our data show the pH 6 citrate buffer solution produced robust plaque regrowth and glycolytic activity, and in fact it was selected as the negative control. The plaque response of the pH 6 buffer system significantly improved with the addition of stannous fluoride, although relative to the stannous fluoride-citrate pH 3.8 system, bulk precipitation of Sn^4+^ species (e.g., Sn(OH)_4_ [40]) is expected to occur with minimal amounts of antimicrobial stannous ions (i.e., Sn^2+^) remaining. The significant difference obtained between the pH 3.8 and 6 citrate buffers in both glycolysis and plaque growth demonstrate the sensitivity of this GAPR method to buffer systems even without potential antimicrobial agents. The inclusion of stannous fluoride in each of the buffer systems produced significant improvements in antiplaque potential, with the pH 3.8 conferring better performance due to the chemistry favoring Sn^2+^. The purpose of the citrate and stannous fluoride experiment is to also show this is one way the GAPR method can be used in the design of potential antiplaque formulations.

The strong response of the two Listerine^®^ mouthwashes in this evaluation reflects the well-known and well-reported clinical performance of Listerine^®^ essential oil mouthwashes [24,42,43,44,45,46], and demonstrates this model’s sensitivity to essential oils. Even earlier than the evaluations of Dr. Miller [47], Listerine was among the first mouthwashes available to the public, though originally, the formula was not intended for oral care primarily but for other personal hygiene purposes [48]. The combination of thymol, menthol, methyl salicylate and eucalyptol in an alcohol-based formulation has cemented its place in the list of FDA-approved antiplaque/antigingivitis agents [15]. The results from our PGRM evaluation echoes another [7], whereby essential oils seem to provide better antigrowth benefits than antiglycolysis benefits. We note that different essential oils, and combinations of essential oils with other agents, may give rise to novel antiplaque formulations exhibiting promising antiglycolysis and antigrowth performance.

Separately, one of the primary reasons to add zinc to oral care formulations is to combat oral malodor, otherwise known as halitosis [49,50,51]. Studies have shown that zinc salts, including zinc chloride which is what we evaluated here, functions largely as a bacteriostatic and impairs the microbial catabolism of peptides [49,51,52]. Volatile gases, which are produced through catabolism of proteins and are the hallmark of halitosis, are not typically neutralized directly by zinc salts (including zinc chloride); rather, sodium chlorite solutions, which evolve chlorine dioxide gas, are used [50]. As synergistic effects are observed when zinc salts are combined with other active agents, including triclosan, CPC, other zinc salts, essential oils, etc. [5,51], there is strong interest in the performance of multiagent formulations [24,53,54]. Our investigations on simple zinc chloride solutions sought to probe the plaque response to only a highly water-soluble zinc salt, and the data suggest zinc salts are more likely to deliver antigrowth benefits relative to antiglycolysis benefits. Although we utilized gingival plaque (which typically has lower populations of microbes responsible for oral malodor, these results are consistent with zinc–bacteria observations and appear to mirror clinical outcomes [51,55]. Additionally, future experiments focused on halitosis treatments could be performed by harvesting plaque from the tongue (instead of the gingival region). Using pH and optical density measurements to gauge the quality of plaque response against negative and positive controls, internal evaluations revealed this process could be repeated for at least five days but begins to diminish after around 10 days; as such, we recommend harvesting fresh plaque on a weekly basis.

The observation of negative percent changes in regrowth indicates the plaque sample density was reduced at four hours relative to baseline. Because the same volume was dispensed in each microwell (i.e., 200 μL), the decrease in optical densities could be explained by any of the following either alone or in combination: microbial death, whereby the dead organisms were inherently less dense relative to surviving organisms’ density; catabolism of dead organisms by live organisms; and/or changes in growth–death rate, whereby ‘new’ organisms may be much less dense than original live or dead organisms. Based on our experiences in using this model on a variety of agents and formulations, we believe the most likely explanation resides in cell death and/or catabolism [56]. Regardless of the reason, the data are not meant to be absolute numbers but should be taken in context relative to the other control groups in the experiments.

Importantly, we note this GAPR method is designed to probe potential chemotherapeutic modes of action and is not designed to test the merit of physical removal of plaque. To show method versatility, the present evaluations probed different antiplaque modes of action including metal ion coordination (Sn^2+^), ionic (F^−^), ionic + lipophilic (CPC), lipophilic (essential oils), and metal ion coordination plus oxidation (ZnCl_2_). Among these evaluations, the CPC groups effected strong antiplaque performance, reflecting the dual-action potency afforded by its chemical structure. Given that present-day formulations have multiple ‘active’ ingredients, this method may likely find use in the research, development, or quality-control assessments of multi-action oral care formulations.

The limitations of this lab model include the fact that it is a lab model and does not guarantee clinical success and is not a surrogate to well-designed clinical evaluations. Additionally, some agents or formulations may demonstrate antiplaque potential through a physical removal means: this model cannot readily assess those mechanisms. Other limitations are that this model may not reflect clinically grown biofilms, and is unable to distinguish between commensal and pathogenic species; rather, plating and counting is recommended. Thus, the model could be coupled with other techniques to more fully understand the microbial characterization and response.

It remains challenging to screen novel agents or formulations because inhibition or kill-time assays, which typically provide critical insight into a small subset of microorganisms, may or may not prove out in the clinical setting for various reasons (e.g., bioavailability, retention, plaque matrix effects, etc.). As such, PGRM models may help bridge standard lab-based biological screens and clinics; additionally, they might strengthen scientific understanding, which, in turn, helps shape marketing claims. As an example, the short discussion below, which is not meant to be disparaging to Wrigley^®^ (i.e., this company is a stout supporter of oral health and markets effective products towards that end), helps illustrate the scientific, marketing, and business risks that exist for formulations with planned commercial expectations.

In the mid-2000’s, Wrigley^®^ conducted a series of evaluations on the promising antimicrobial benefits of magnolia bark extract. The bulk of the lab-based antimicrobial work relied on inhibition and kill-time assays on single organisms, as well as some small-scale clinical screenings analyzing the volunteers’ saliva [5,6]. However, when the Eclipse^®^ gum containing the magnolia bark extract was commercialized, litigation over unsubstantiated antimicrobial claims ensued, and the US National Advertising Review Board ruled that, in view of the conducted studies (lab and clinical screenings), the antimicrobial marketing claims were too aggressive [57]. Ultimately, the product was pulled and further use of the magnolia bark extract ceased.

While PGRM models are most often used on formulations containing CPC or SnF_2_, we have shown the applicability of our GAPR method to those agents and others, including NaF, ZnCl_2_, and Listerine^®^ essential oil mouthwash with and without alcohol. Although not shown here, we routinely use commercially marketed SnF_2_ or CPC systems (including Procter & Gamble, Haleon, and Colgate SnF_2_ toothpastes and CPC mouthwashes), as well as various essential oil mouthwashes (e.g., Listerine^®^), as positive controls in the screening of agents and formulations for our clients. Although we used duplicates in the present assessments, naturally, this model can be adjusted to accommodate additional replicates as desired. Ideally, results gleaned from this GAPR model would contribute to mechanism of action understanding, support quality control efforts for new and/or existing formulations, or help provide critical information on whether to advance to clinical evaluations.

## 5. Conclusions

Well-designed and executed clinical studies may not be feasible or practical in the screening of promising antiplaque agents or formulations. But antiplaque lab models often rely on inhibition or kill-time assays which may not provide sufficient evidence to warrant strong marketing or scientific claims. To help expand lab evaluation methodologies, an in vitro PGRM model was developed for the evaluation of a variety of systems including CPC, NaF, SnF_2_, ZnCl_2_, and commercially available Listerine^®^ essential oil formulations with and without alcohol. While certainly not a substitute for clinical evaluations, our lab results appear consistent with clinical outcomes and show the use of this GAPR model might be used as a quality and/or screening device in the evaluation of putative agents and formulations.

## Figures and Tables

**Table 1:** Antimicrobial performance of aqueous CPC solutions in terms of glycolysis (i.e., acid production), acid inhibition percentage, and plaque regrowth. Large negative glycolysis and/or large plaque regrowth values indicate poor antimicrobial potential.

Treatment	Glycolysis (% pH) *	Acid Inhibition (%) **	Plaque Regrowth (% OD) ***
0.1% CPC	−0.4 (0.2) ^a^	98.8	−0.9 (1.6) ^A^
0.07% CPC	−1.1 (0.1) ^b^	96.5	14.9 (1.7) ^B^
0.05% CPC	−11.8 (0.1) ^c^	61.3	14.6 (3.8) ^B^
0.03% CPC	−22.5 (0.1) ^d^	26.2	55.3 (2.0) ^C^
0.01% CPC	−28.4 (0.3) ^e^	6.6	81.7 (1.9) ^D^
0.001% CPC	−30.4 (0.0) ^f^	0.0	79.0 (1.2) ^D^

* Lowercase letter superscripts (a, b, etc.) for mean (SEM) glycolysis data (from Equation (1)) denote significant differences (*p* < 0.05) with a > b, etc. within this column. ** Acid inhibition was calculated using Equations (1) and (2) and serves as an efficiency parameter to estimate relative antiglycolysis potential. The 0.001% CPC treatment was selected as the reference negative control. *** Uppercase letter superscripts (A, B, etc.) for mean (SEM) plaque regrowth data (from Equation (3)) denote significant differences (*p* < 0.05) with A < B, etc. within this column.

**Table 2:** Antimicrobial performance of aqueous NaF solutions in terms of glycolysis (i.e., acid production), acid inhibition percentage, and plaque regrowth.

Treatment	Glycolysis (% pH) *	Acid Inhibition (%) **	Plaque Regrowth (% OD) ***
0.1% CPC	−2.2 (0.0) ^a^	93.1	−7.0 (5.4) ^B^
22,500 ppm F	−12.3 (0.4) ^b^	60.8	−20.8 (2.1) ^A^
5000 ppm F	−20.1 (0.0) ^c^	36.2	2.4 (1.5) ^B^
1000 ppm F	−26.6 (0.1) ^d^	15.5	100.7 (0.7) ^C^
100 ppm F	−30.0 (0.0) ^e^	4.8	161.0 (5.7) ^E^
1 ppm F	−30.3 (0.1) ^e^	3.8	160.3 (3.9) ^E^
0.1 ppm F	−30.1 (0.1) ^e^	4.6	160.6 (1.6) ^E^
0.001% CPC	−31.5 (0.0) ^f^	0.0	113.1 (2.2) ^D^

* Lowercase letter superscripts (a, b, etc.) for mean (SEM) glycolysis data (from Equation (1)) denote significant differences (*p* < 0.05) with a < b, etc. within this column. ** Acid inhibition was calculated using Equations (1) and (2) and serves as an efficiency parameter to estimate relative antiglycolysis potential. The 0.001% CPC treatment was selected as the reference negative control. *** Uppercase letter superscripts (A, B, etc.) for mean (SEM) plaque regrowth data (from Equation (3)) denote significant differences (*p* < 0.05) with A < B, etc. within this column.

**Table 3:** Antimicrobial performance of citrate buffer (CB) solutions (pH 3.8 or 6) with or without SnF_2_ in terms of glycolysis (i.e., acid production), acid inhibition percentage, and plaque regrowth. Large negative glycolysis and/or large plaque regrowth values indicate poor antimicrobial potential.

Treatment	Glycolysis (% pH) *	Acid Inhibition (%) **	Plaque Regrowth (% OD) ***
CB3.8 + 0.4% SnF_2_	−7.0 (0.4) ^a^	72.4	26.1 (1.7) ^A^
CB3.8	−11.7 (0.1) ^b^	53.7	88.4 (2.5) ^B^
CB6 + 0.4% SnF_2_	−18.8 (0.1) ^c^	25.4	91.0 (3.9) ^B^
CB6	−25.2 (0.2) ^d^	0.0	145.7 (4.5) ^C^

* Lowercase letter superscripts (a, b, etc.) for mean (SEM) glycolysis data (from Equation (1)) denote significant differences (*p* < 0.05) with a > b, etc. within this column. ** Acid inhibition was calculated using Equations (1) and (2) and serves as an efficiency parameter to estimate relative antiglycolysis potential. The citrate buffer pH 6 (CB6) treatment was selected as the reference negative control. *** Uppercase letter superscripts (A, B, etc.) for mean (SEM) plaque regrowth data (from Equation (3)) denote significant differences (*p* < 0.05) with A < B, etc. within this column.

**Table 4:** Antimicrobial performance of Listerine^®^ essential oil mouthwashes with (A. Ultraclean^®^) or without (B, Original^®^ Cool Mint) alcohol in terms of glycolysis (i.e., acid production), acid inhibition percentage, and plaque regrowth. Large negative glycolysis and/or large plaque regrowth values indicate poor antimicrobial potential.

Treatment	Glycolysis (% pH) *	Acid Inhibition (%) **	Plaque Regrowth (% OD) ***
Listerine^®^ A	−11.6 (0.2) ^a^	66.7	−34.7 (4.2) ^A^
Listerine^®^ B	−23.1 (0.1) ^b^	33.8	−2.9 (0.4) ^B^
0.001% CPC or MO ^1^	−34.8 (0.1) ^c^	0.0	80.5 (6.7) ^C^

^1^ In the plaque regrowth assessment, instead of using 0.001% CPC, we used mineral oil (MO) as the negative control. * Lowercase letter superscripts (a, b, etc.) for mean (SEM) glycolysis data (from Equation (1)) denote significant differences (*p* < 0.05) with a > b, etc. within this column. ** Acid inhibition was calculated using Equations (1) and (2) and serves as an efficiency parameter to estimate relative antiglycolysis potential. The 0.001% CPC treatment was selected as the reference negative control. *** Uppercase letter superscripts (A, B, etc.) for mean (SEM) plaque regrowth data (from Equation (3)) denote significant differences (*p* < 0.05) with A < B, etc. within this column.

**Table 5:** Antimicrobial performance of aqueous ZnCl_2_ solutions in terms of glycolysis (i.e., acid production), acid inhibition percentage, and plaque regrowth. Large negative glycolysis and/or large plaque regrowth values indicate poor antimicrobial potential.

Treatment	Glycolysis (% pH) *	Acid Inhibition (%) **	Plaque Regrowth (% OD) ***
0.1% CPC	−4.4 (0.0) ^a^	86.4	4.1 (1.0) ^A^
0.2% ZnCl_2_	−22.2 (0.0) ^b^	31.4	15.0 (4.6) ^A^
0.08% ZnCl_2_	−25.3 (0.2) ^c^	21.8	33.7 (4.5) ^B^
0.001% CPC	−32.4 (0.1) ^d^	0.0	70.4 (0.1) ^C^

* Lowercase letter superscripts (a, b, etc.) for mean (SEM) glycolysis data (from Equation (1)) denote significant differences (*p* < 0.05) with a > b, etc. within this column. ** Acid inhibition was calculated using Equations (1) and (2) and serves as an efficiency parameter to estimate relative antiglycolysis potential. The 0.001% CPC treatment was selected as the reference negative control. *** Uppercase letter superscripts (A, B, etc.) for mean (SEM) plaque regrowth data (from Equation (1)) denote significant differences (*p* < 0.05) with A < B, etc. within this column.

## Data Availability

Data presented in this are available upon written request.
